# Development and Validation of an AI‐Assisted Predictive Model Integrating R2* Mapping and Clinical Indicators for Clinically Significant Prostate Cancer

**DOI:** 10.1002/cam4.71656

**Published:** 2026-02-18

**Authors:** Xin Li, Yonggui Shi, Jing Fang, Rong Zhang, Xiaojing He, Guangyong Ai

**Affiliations:** ^1^ Department of Radiology The Second Affiliated Hospital of Chongqing Medical University Chongqing China

**Keywords:** artificial intelligence, biparameter, nomogram, prostate cancer, prostate imaging reporting and data system, R2* mapping

## Abstract

**Background:**

Limited evidence exists on the diagnostic performance of Artificial Intelligence (AI)‐assisted Simplified Prostate Imaging Reporting and Data System version 2.1 (S‐PI‐RADS v2.1) combined with quantitative MRI parameters for detecting clinically significant prostate cancer (csPCa).

**Purpose:**

To develop and validate a nomogram incorporating AI‐assisted S‐PI‐RADS v2.1 (based on biparametric MRI [bpMRI]) and R2* mapping for csPCa prediction.

**Methods:**

This prospective study enrolled 345 patients grouped by pathology: non‐csPCa with benign prostatic hyperplasia (*n* = 230) and csPCa (*n* = 115). Clinical (age, body mass index [BMI], prostate‐specific antigen [PSA], free PSA) and imaging parameters (prostate volume [PV], S‐PI‐RADS score, R2*) were analyzed. Independent predictors were identified via logistic regression. A nomogram was developed using R software with the DynNom package (Version 2.0) and validated (1000 bootstrap iterations), with performance assessed by area under the curve (AUC), calibration, decision curve analysis (DCA), and DeLong test (*p* < 0.05 significant).

**Results:**

Independent csPCa predictors included BMI, PSA ≥ 10 ng/mL, PV, S‐PI‐RADS scores 4–5, and R2* (all *p* < 0.05). The full model (BMI + PSA + PV + S‐PI‐RADS + R2*) showed superior discrimination (AUC = 0.915) versus the baseline model (AUC = 0.891, *p* = 0.008), with 85.2% sensitivity and 80.9% specificity. Internal validation was robust (C‐index = 0.884). DCA confirmed clinical utility. An interactive nomogram was deployed (https://aiguangyong2025.shinyapps.io/dynnomapp/).

**Conclusion:**

The AI‐enhanced nomogram integrating clinical and multiparametric MRI data accurately predicts csPCa noninvasively, with R2* significantly improving performance. This tool facilitates personalized clinical decision‐making.

## 1 Introduction

Prostate cancer (PCa) is one of the most common malignancies and the second leading cause of cancer‐related death in men [1]. The treatment and prognosis of PCa vary widely to its histological subtypes. Tumors with high metastatic potential often have a poor prognosis. Thus, early diagnosis of csPCa is of great clinical significance. In csPCa, glandular structure destruction, increased cellularity, and reduced extracellular space occur. It is highly aggressive, fast‐progressing, and has a poor prognosis, requiring active clinical intervention. In contrast, non‐clinically significant (ncsPCa), including benign prostatic hyperplasia, is well‐differentiated, with normal glandular structure and cell spacing. It has low malignancy, weak invasiveness, and slow progression. For ncsPCa, only follow‐up and active surveillance are needed, as overtreatment may burden patients and reduce their quality of life [2]. Invasive needle biopsy remains the current “gold standard” for the accurate diagnosis of prostate cancer, but it is prone to complications such as bleeding, fever, prostatic abscess, and sepsis. Therefore, the exploration of non‐invasive diagnostic methods has become a research hotspot in recent years.

Additionally, artificial intelligence (AI) advancements, particularly radiomics and deep learning (DL), are transforming clinical diagnosis and treatment planning. These technologies can automatically segment and detect prostate cancer lesions, accurately diagnose csPCa and predict poor pathological prognosis of PCa [3, 4, 5]. Currently, while AI model‐based data analysis can demonstrate the relationship between medical images and clinical outcomes, it often lacks biological interpretability [6]. PI‐RADS v2.1 based on multiparametric MRI (mp‐MRI) is a set of guidelines for a comprehensive assessment of PCa, and studies have confirmed its diagnostic efficacy for csPCa [7, 8]. Although the European Society of Urogenital Radiology (ESUR) recommends the use of dynamic contrast‐enhanced (DCE) imaging in its guidelines, controversies persist [9]. Therefore, bp‐MRI protocols consisting of T2‐weighted imaging (T2WI) and diffusion‐weighted imaging (DWI) sequences have become a research hotspot in recent years [10]. Simplified bp‐MRI matches mp‐MRI in PCa diagnostic performance while overcoming mp‐MRI's high costs, long scan times, and contrast agent risks [11]. However, PI‐RADS assessments require a high degree of expertise and suffer from limited consistency due to persistent inter‐reader variability [12, 13]. Researchers are addressing these challenges through emerging quantitative MRI methods, predictive models incorporating clinical data, and the development of sophisticated AI systems [3, 14].

R2* mapping is a reliable non‐invasive MRI technique for quantifying tissue iron content. Iron deposition shortens T2* via paramagnetic effects, thereby influencing tissue R2*. Cellular iron metabolism is often closely associated with prostate cancer progression, playing a critical role in angiogenesis and tumor metastasis [15]. R2* mapping exhibits extremely high sensitivity in detecting changes in the local magnetic environment caused by iron content variations in biological tissues [16]. It enables non‐invasive acquisition of objective quantitative indicators, providing additional lesion information from the perspective of the tumor microenvironment [17, 18, 19].

To address these critical gaps: while bp‐MRI combined with S‐PI‐RADS simplifies csPCa diagnosis, inter‐reader variability from subjective assessment, poor AI model interpretability, and inadequate integration of tumor morphological and microenvironmental features limit accurate csPCa‐ncsPCa differentiation. We hypothesize that AI‐driven S‐PI‐RADS improves assessment consistency and efficiency, and R2* mapping provides objective microenvironmental data to complement morphological assessment; their integration with clinical indicators will significantly enhance csPCa differential diagnostic efficacy and model interpretability.

This study aims to construct a non‐invasive csPCa prediction model integrating AI‐driven S‐PI‐RADS, R2* mapping parameters and clinical indicators, to clarify R2* mapping's value in reducing S‐PI‐RADS subjectivity, verifying diagnostic performance, and enhancing AI model interpretability, thus providing a novel tool for precise non‐invasive csPCa diagnosis.

## 2 Materials and Methods

### 2.1 Patients

**FIGURE 1:**
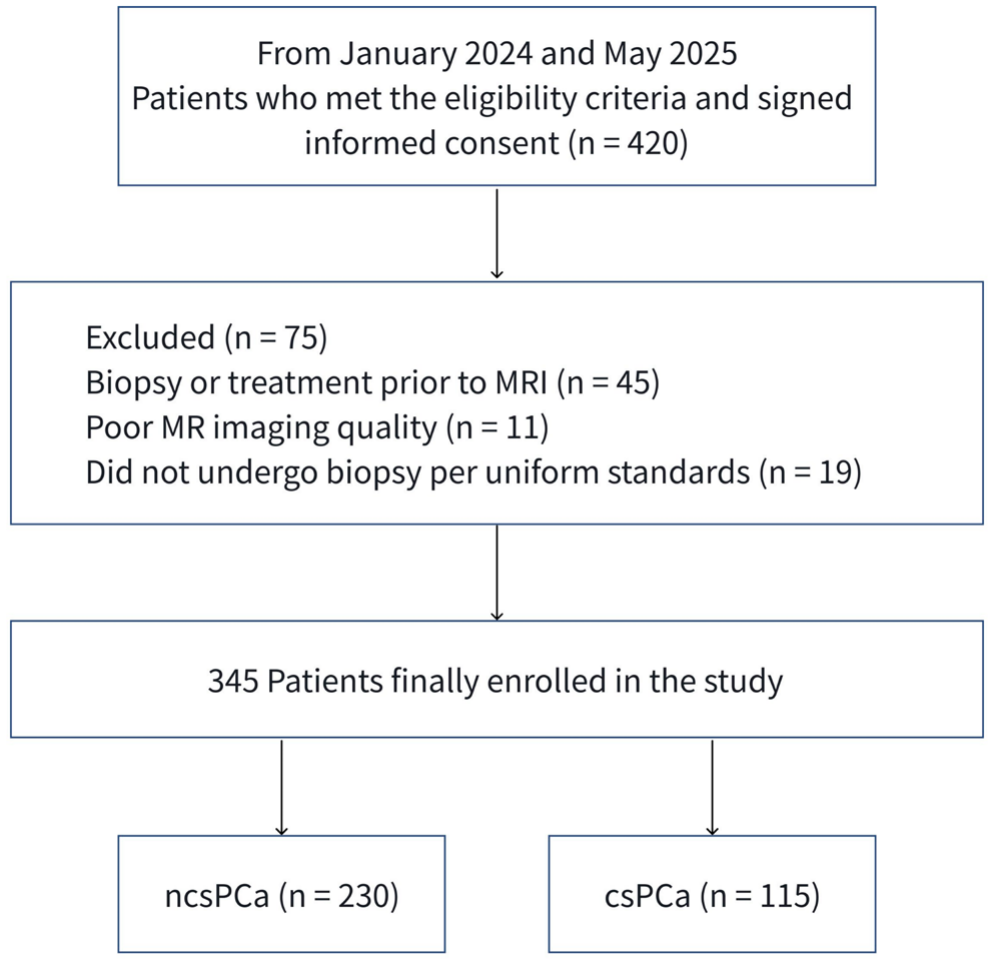
Flow diagram of the study population. csPCa, clinically significant prostate cancer; ncsPCa, clinically insignificant prostate cancer or benign prostatic hyperplasia.

The study was approved by the hospital's Ethics Committee (Approval No. [2024]130). A total of 420 patients with suspected prostate cancer admitted to the Second Affiliated Hospital of Chongqing Medical University between January 2024 and May 2025 were prospectively enrolled in this study. Written informed consent was obtained from all patients. All patients underwent mp‐MRI and R2* mapping scans before surgery. Inclusion criteria were (1) clinical suspicion of PCa (elevated PSA or positive digital rectal examination); (2) all patients provided informed consent; (3) all patients were pathologically diagnosed with PCa or benign prostatic hyperplasia (BPH) via ultrasound‐guided transrectal biopsy or surgical pathology, and all underwent prostate MRI (including T2WI, DWI, and T2* mapping sequences) within 1 month before biopsy. The exclusion criteria were (1) any contraindications to MRI examination; (2) poor image quality or incomplete imaging sequences that prevented analysis; (3) history of prior prostate biopsy or treatment (hormone therapy, radiotherapy, chemotherapy, or surgery); and (4) other diseases affecting iron metabolism except PCa. A total of 345 patients were finally included in the study, and the patient selection process is shown in Figure 1.

### 2.2 Clinical Data Collection

Patient age, BMI, PSA (stratified into three groups: < 4, 4–10, and ≥ 10), PV, fPSA, S‐PI‐RADS score (stratified into four groups: ≤ 2, 3, 4, and 5 points), biopsy and/or surgical pathology results, and lesion location were collected through retrieval of the electronic medical record system. Within 1 month of completing MRI examinations, all 345 patients underwent transrectal ultrasound (TRUS)‐guided 12‐core systematic biopsy. All pathological specimens were reviewed, and the Gleason scores (GS) were assigned according to the 2014 consensus guideline of the International Society of Urological Pathology (ISUP) by a pathologist with 10 years of clinical experience.

According to the results, the study subjects were divided into a csPCa group and a ncsPCa group: clinically significant PCa was defined as GS ≥ 7 (including cases with a 3 + 4 pattern where Gleason 4 is prominent but not predominant) and/or tumor diameter ≥ 5 mm, comprising 115 cases; the ncsPCa group included BPH with or without chronic inflammation and tumors with Gleason scores < 7, totaling 230 cases. Among them, there were 203 cases of BPH and 27 cases of PCa with Gleason scores < 7.

### 2.3 Imaging Acquisition

The MRI examinations were performed on a 3.0 T scanner (Magnetom Prisma, Siemens Healthineers, Erlangen, Germany) with a 18‐channel body phased array coil and combined with a 32‐channel spinal coil. The routine scanning sequences included T1‐weighted imaging (T1WI), noncompacted fat T2WI, fat‐suppressed T2WI, and DWI. The scanning parameters for transverse T2WI were as follows: repetition time/echo time (TR/TE): 3290/77 ms; field of view (FOV): 200 mm × 200 mm; resolution: 0.5 mm × 0.5 mm × 3 mm; slice thickness 3 mm; slice gap 0 mm; and two‐signal averages. DWI scan parameters: TR/TE: 3800/52, 84 ms; FOV: 200 mm × 200 mm; resolution: 1.7 mm × 1.7 mm × 3 mm; slice thickness 3 mm; slice gap 0 mm; b‐values 0, 1400 s/mm^2^; and averages 1/2. The R2* mapping images were obtained using a T2*‐corrected 3D multi‐echo Dixon (q‐Dixon) sequence and the parameters were as follows: TR/TE: 9.14/1.28, 2.53, 3.78, 5.03, 6.28, and 7.53 ms; FOV: 250 mm × 200 mm; matrix size: 160 × 128 × 80; resolution: 0.8 mm × 0.8 mm × 3 mm; slice thickness 3 mm; slice gap 0 mm; flip angle: 4°; and acquisition time of 12 s. The localization planes of the T2WI, DWI, and R2* mapping sequences were aligned as consistently as possible. All parameters are shown in Table S1.

### 2.4 S‐PI‐RADS Categories bpMRI‐Based

The images acquired from bpMRI scans were transferred to the Computer‐Aided Analysis System for Prostate MRI (uAI Discover‐ProstateMR, Shanghai United Imaging Intelligence Medical Technology Co. Ltd.) for post‐processing analysis. This AI system is trained on a large‐scale multi‐center dataset, and its technical principles, performance metrics, and validation methods are consistent with the standards verified in recent high‐impact studies [20, 21]. Glands and cancerous lesions were segmented using deep learning to automatically identify suspicious lesions and assign SI‐PI‐RADS grades. All AI‐generated S‐PI‐RADS scores were independently reviewed and verified by two senior radiologists in our department with over 10 years of professional experience in prostate MRI interpretation. Any discrepancies in scoring were resolved through a consensus review to ensure clinical reliability and consistency. The scoring criteria for bp‐MRI are shown in Table S2. Meanwhile, the maximum superior–inferior diameter (SI), maximum anterior–posterior diameter (AP), and maximum left–right diameter (LR) of the prostate are automatically identified on T2WI to calculate PV automatically [PV (cm^3^) = SI (cm) × AP (cm) × LR (cm) × 0.52] [22].

### 2.5 Image Analysis

**FIGURE 2:**
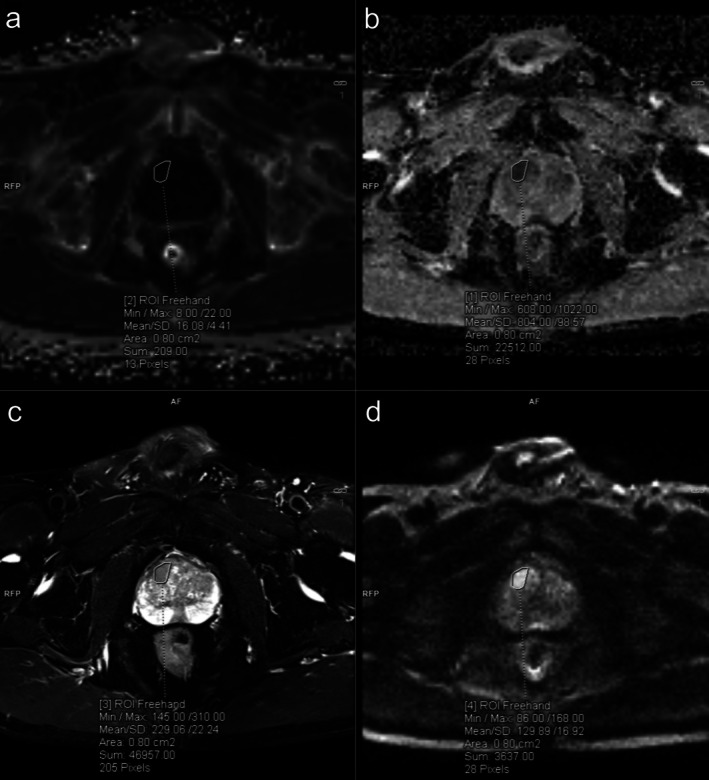
Schematic diagram of ROI selection. (a) R2* mapping; (b) ADC map; (c) T2WI_fs image; (d) DWI image. ADC, apparent diffusion coefficient; DWI, diffusion‐weighted imaging; ROI, regions of interest; T2WI_fs, T2 weighted imaging with fat suppression.

R2* mapping images were automatically generated by the scanner and processed using a commercial software workstation system (Syngo.via, VB60S_HF02, Siemens Healthineers, Germany). Two MRI diagnosticians with 8 years of experience evaluated and analyzed the conventional MRI and R2* mapping images of this cohort. Lesion locations were determined based on detailed pathological records, and the optimal slice showing the lesion was selected by combining T2WI and DWI images. Regions of interest (ROIs) were drawn on T2WI or DWI images according to the morphology and size of the lesions, with an ROI area of approximately 50–150 mm^2^. The ROI drawing avoided the urethra, hemorrhage, and necrotic areas, maintaining a certain distance from the lesion margin to avoid partial volume effects while including most of the lesion area. For R2* mapping, the ROIs from T2WI or DWI were copied and pasted to the same anatomical location, and R2* values were recorded (Figure 2). When patients had multiple lesions, only the lesion with the highest GS or the largest size (if GS was the same) was evaluated. The two radiologists discussed and negotiated differing observations to reach a consensus conclusion.

### 2.6 Implementation of a Dynamic Online Nomogram

The final multivariate logistic regression model was used to construct a dynamic prediction tool. We employed the DynNom package (Version 2.0) in R software (Version 4.2.2) to develop an interactive web application. This package converts the logistic regression model object into a web application via the Shiny framework. The predicted variable values input by users are used to calculate the predicted probability in real time through the model's underlying logistic formula (logit (*p*) = *β*_0_ + *β*_1_ *X*_1_ + ⋯ + *β*_n_ *X*_n_), which is dynamically presented in both graphical and numerical forms. The core construction formula of this study is: Logit (P (csPCa)) = −9.056 + 0.175 × R2* + 0.093 × BMI + 2.301 × PSA_group ≥ 10 + 0.606 × PSA_group4–10 + −0.027 × PV + 1.309 × S‐PI‐RADS 3 + 2.935 × S‐PI‐RADS 4 + 3.838 × S‐PI‐RADS 5.

### 2.7 Statistical Analysis

Statistical analyses and graphical representations were conducted utilizing SPSS version 26.0 (IBM), and R software version 4.2.2. Categorical variables were expressed as counts and percentages. Continuous variables were expressed as mean ± standard deviation (SDs) if normally distributed, based on visual inspection and the Shapiro–Wilk test. Otherwise, categorical variables were expressed as median with IQRs. Differences between patients with csPCa or ncsPCa were assessed for significance using the *Chi*‐square test in the case of categorical data, or using the Welch Two Sample *t*‐test or Wilcoxon rank‐sum test in the case of continuous data. Potential associations between S‐PI‐RADS and ISUP were explored using Spearman's correlation.

To mitigate potential overfitting risks, all candidate variables in this study (including age, BMI, PSA groups, PV, S‐PI‐RADS score, and R2* value) were pre‐specified. Variable selection was based on clinical prior knowledge and evidence from previous literature. A total of 115 cases of the outcome event (csPCa) were included, and six variables were finally incorporated into the multivariate logistic regression model. The event per variable (EPV) ratio was approximately 19:1, which is far higher than the recommended threshold of 10:1. Univariate and multivariate logistic regression were performed to screen for independent risk factors of csPCa. A nomogram for predicting the risk of csPCa was developed based on these independent risk factors. Meanwhile, internal validation of the model was tested using 1000 bootstrap resamples, and the calibration plot was shown graphically.

The area under the curve (AUC) of the receiver operating characteristic (ROC) was employed to assess the discrimination of the model, employing the optimal cutoff derived from the Youden index. AUC differences were evaluated using the DeLong test. The calibration curve assessed the model's calibration. The clinical utility of the model was evaluated using decision curve analysis (DCA). All *p* values were two‐sided, and differences were considered statistically significant at *p* < 0.05.

## 3 Result

### 3.1 Demographic and Clinical Characteristics

The study population included 345 patients, among whom 115 had csPCa, 230 had ncsPCa (203 had BPH and 27 had PCa with Gleason scores < 7). Age and BMI were comparable between the csPCa and ncsPCa groups. The csPCa group had significantly higher median PSA levels (8 vs. 53, *p* < 0.001), median fPSA levels (1 vs. 4, *p* < 0.001), and a significantly higher proportion of patients with PSA ≥ 10 (81.7% vs. 42.2%) compared to the ncsPCa group. There was a significant difference in the distribution of S‐PI‐RADS scores (*p* < 0.001), with 65.2% of the csPCa group assigned a score of 5, while the ncsPCa group predominantly had score 3 (48.7%). Patients with csPCa also showed significantly higher R2* and lower PV (*p* < 0.001).

The demographic and clinical characteristics of the 345 included patients are detailed in Table 1.

**TABLE 1:** Demographic and clinical characteristics of enrolled patients.

Characteristic	ncsPCa (*n* = 230)	csPCa (*n* = 115)	*p*
Age (year)	71 (64, 75)	72 (67, 77)	0.109^a^
BMI (kg/m^2^)	23.34 ± 2.82	22.91 ± 3.48	0.248^b^
R2* (s^−1^)	17 (15, 20)	22 (18, 27)	< 0.001^a^
PSA (ng/mL)	8 (5, 14)	53 (13, 300)	< 0.001^a^
PSA_group (ng/mL)			< 0.001^c^
< 4	45 (19.6%)	8 (7.0%)	
4–10	88 (38.3%)	13 (11.3%)	
≥ 10	97 (42.2%)	94 (81.7%)	
PV (cm^3^)	61 (43, 84)	45 (30, 57)	< 0.001^c^
fPSA (ng/mL)	1 (1, 2)	4 (2, 25)	< 0.001^c^
S‐PI‐RADS			< 0.001^c^
2	39 (17.0%)	1 (0.9%)	
3	112 (48.7%)	18 (15.7%)	
4	45 (19.6%)	21 (18.3%)	
5	34 (14.8%)	75 (65.2%)	

*Note:* Data are represented in Median (Q1, Q3), mean ± SD or frequency (%). *p* value < 0.05 indicates a significant difference between the two groups.

Abbreviations: BMI, body mass index; csPCa, clinically significant prostate cancer; fPSA, free PSA; ncsPCa, non‐clinically significant prostate cancer; PSA, serum prostatespecific antigen; PV, prostate volume; S‐PI‐RADS, simplified prostate imaging reporting and data system.

^a^ Wilcoxon rank‐sum test.

^b^ Welch Two Sample *t*‐test.

^c^ *Chi*‐squared test.

### 3.2 Univariate and Multivariate Logistic Regression Analyses

The results of the univariate and multivariate analysis are summarized in Table 2.

**TABLE 2:** Univariate and multivariate analyses of predictors for csPca.

Characteristic	Univariable		Multivariable	
	OR (95% CI)	*p*	OR (95% CI)	*p*
R2* (s^−1^)	1.18 (1.13–1.24)	< 0.001***	1.17 (1.10–1.25)	< 0.001***
Age (years)	1.03 (1.00–1.05)	0.047*	1.03 (0.98–1.07)	0.238
BMI (kg/m^2^)	0.95 (0.89–1.03)	0.215	1.17 (1.03–1.32)	0.016*
PSA_group (ng/mL)				
< 4	Ref.		Ref.	
4–10	0.83 (0.32–2.15)	0.703	1.86 (0.52–6.73)	0.341
≥ 10	5.45 (2.44–12.18)	< 0.001***	6.01 (1.81–20.00)	0.003**
PV (cm^3^)	0.98 (0.97–0.99)	< 0.001***	0.97 (0.96–0.99)	< 0.001***
fPSA (ng/mL)	1.10 (1.06–1.15)	< 0.001***	1.00 (0.96–1.04)	0.932
S‐PI‐RADS				
2	Ref.		Ref.	
3	6.27 (0.81–48.51)	0.079	3.08 (0.37–25.64)	0.299
4	18.20 (2.34–141.57)	0.006**	13.24 (1.53–114.31)	0.019*
5	86.03 (11.35–652.32)	< 0.001***	23.86 (2.82–201.79)	0.004**

Abbreviations: BMI, body mass index; CI, confidence interval; csPCa, clinically significant prostate cancer; fPSA, free PSA; OR, odds ratio; PSA, serum prostate‐specific antigen; PV, prostate volume; Ref, reference; S‐PI‐RADS, simplified prostate imaging reporting and data system.

* *p* < 0.05.

** *p* < 0.01.

*** *p* < 0.001.

Univariate logistic regression analyses showed that age (*p* = 0.047), PSA ≥ 10 (*p* < 0.001), PV (*p* < 0.001), S‐PI‐RADS score of 4 (*p* = 0.006), S‐PI‐RADS score of 5 (*p* < 0.001), fPSA (*p* < 0.001), and R2* (*p* < 0.001) were significant associations with the risk of csPCa.

Multivariate analysis further indicated that BMI (OR = 1.17; 95% CI = 1.03–1.32; *p* = 0.016), PSA ≥ 10 (OR = 6.01; 95% CI = 1.81–20.00; *p* = 0.003), PV (OR = 0.97; 95% CI = 0.96–0.99; *p* < 0.001), S‐PI‐RADS score of 4 (OR = 13.24; 95% CI = 1.53–114.31; *p* = 0.019), S‐PI‐RADS score of 5 (OR = 23.86; 95% CI = 2.82–201.79; *p* = 0.004) and R2* (OR = 1.17; 95% CI = 1.10–1.25; *p* < 0.001) were identified as independent predictors of csPCa. Higher R2*, PSA, and lower PV correlated with a higher csPCa risk.

### 3.3 Development and Validation of a Predictive Model Based on Multivariate Regression Analysis

**FIGURE 3:**
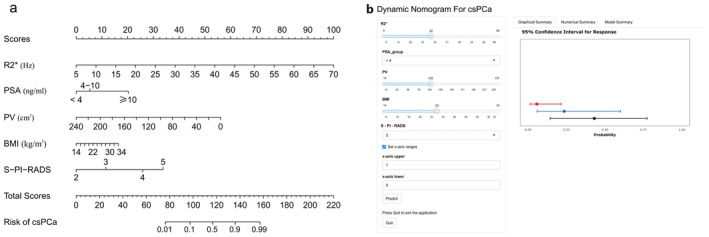
Nomogram for predicting the probability of csPCa. (a) The nomogram was constructed by incorporating the following five variables: R2*, PSA, PV, BMI, and S‐PI‐RADS. Higher total points indicated a higher prevalence for csPCa. (b) The online dynamic nomogram can be accessed at https://aiguangyong2025.shinyapps.io/dynnomapp/. BMI, body mass index; csPCa, clinically significant prostate cancer; PSA, serum prostate‐specific antigen; PV, prostate volume; S‐PI‐RADS, simplified prostate imaging reporting and data system.

**FIGURE 4:**
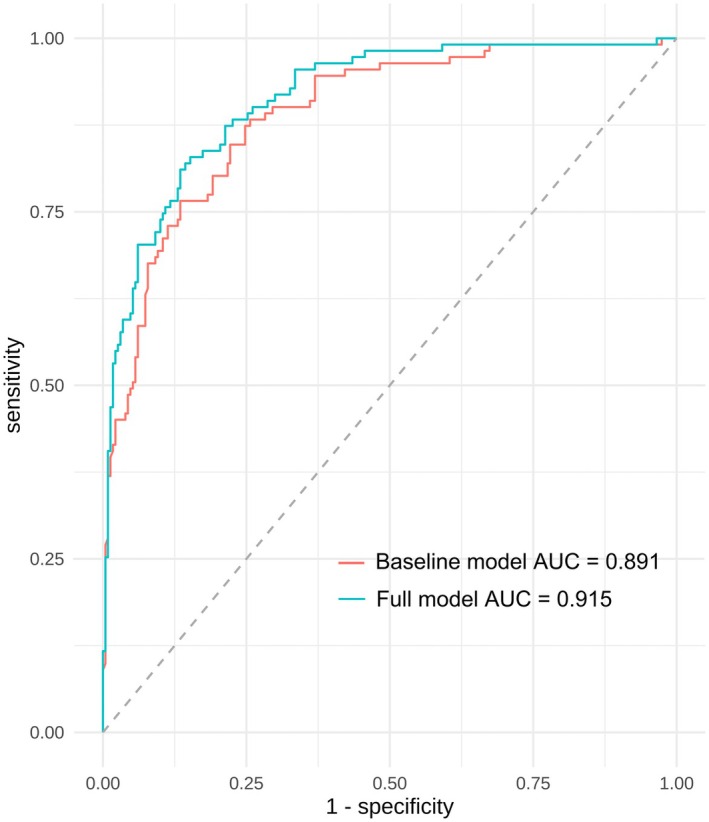
Receiver operating characteristic curves of the baseline model (BMI, PSA, PV, S‐PI‐RADS) and full model (R2*, BMI, PSA, PV, S‐PI‐RADS). AUC, area under the curve; BMI, body mass index; PSA, prostate‐specific antigen; PV, prostate volume; S‐PI‐RADS, prostate imaging reporting and data system.

Based on the results of the multivariate logistic regression analysis, two predictive models were developed: a baseline model with predictors including BMI, PSA, PV, and S‐PI‐RADS; and a full model that additionally incorporated R2*, illustrated in a nomogram (Figure 3A). A web‐based dynamic online nomogram was also created (https://aiguangyong2025.shinyapps.io/dynnomapp/) (Figure 3B). The ROC curves analysis demonstrated that incorporating R2* significantly enhanced the model's predictive performance for csPCa. Specifically, the AUC increased from 0.891 (95% CI 0.837–0.914, *p* < 0.001) to 0.915 (95% CI 0.875–0.937, *p* < 0.001) when R2* was added to the predictive model (Figure 4). At the optimal threshold of 30.7%, the full model demonstrated a sensitivity of 85.2% and a specificity of 80.9%, significantly outperforming the baseline model (*p* = 0.008).

**FIGURE 5:**
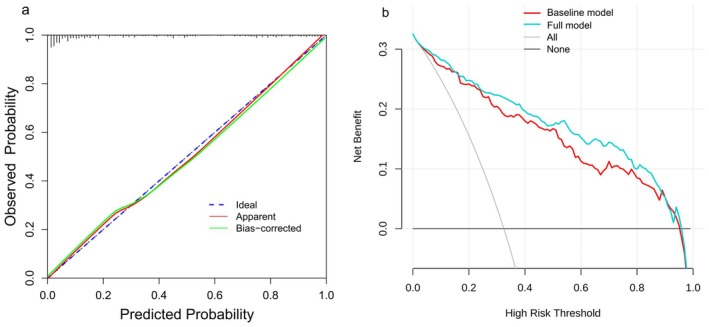
Valuation and assessment of full model. (a) Calibration curve of the nomogram. The apparent curve represents the relationship between predicted probability and actual probability of clinically significant prostate cancer based on our entire study population. The bias‐corrected curve was plotted by bootstrapping using 1000 resamples. The ideal curve is the 45° line which means perfect prediction. (b) The DCA curve of the baseline model (BMI, PSA, PV, S‐PI‐RADS) and full model (R2*, BMI, PSA, PV, S‐PI‐RADS). BMI, body mass index; DCA, decision curve analysis; PSA, prostate‐specific antigen; PV, prostate volume; S‐PI‐RADS, prostate imaging reporting and data system.

For the full model, internal validation using the Bootstrap method (1000 resamples) produced a C‐statistic of 0.887, indicating good internal validation. The calibration curve shows that both the predictive model and its adjusted version closely match the ideal curve, indicating good calibration (Figure 5A). DCA was performed for both the baseline model and the full model. Results show that when the threshold probability is between 10% and 75%, both models offer greater net benefit than the “All” or “None” strategies. Importantly, the full model consistently achieves a higher overall net benefit, thereby demonstrating its superior clinical utility (Figure 5B).

## 4 Discussion

In this study, we explored the predictive value of a nomogram constructed using AI‐assisted S‐PI‐RADS v2.1 based on bpMRI, R2* mapping, and clinical indicators for csPCa. Our results showed that the nomogram model integrating BMI, PSA, PV, S‐PI‐RADS, and R2* demonstrated excellent performance in noninvasively predicting csPCa. Notably, the inclusion of R2* significantly enhanced the model's predictive efficacy, providing a more comprehensive and reliable assessment tool for clinical decision‐making.

In recent years, multiple clinical indicators have been applied to improve the diagnosis of PCa. This study showed that BMI, PSA ≥ 10, PV, S‐PI‐RADS score of 4, S‐PI‐RADS score of 5, and R2* were independent predictors of clinically significant cancer, while age, fPSA, and S‐PI‐RADS score of 3 were not. Univariate analysis suggested that age increase might be associated with an elevated risk of csPCa (OR = 1.03, 95% CI: 1.00–1.05, *p* = 0.047). However, in multivariate analysis, similar to reports by Li et al., the effect of age did not reach high significance, possibly due to the attenuation of its independent role after adjusting for other factors in the model [23]. Age as a single factor may not be sufficient to meet clinical needs in terms of diagnostic accuracy and timeliness. Studies have suggested that obesity is a recognized risk factor for multiple cancers, and is associated with the incidence and prognosis of prostate cancer [24]. In the univariate analysis of this study, BMI was not significantly associated with csPCa, but multivariate analysis revealed borderline significance (OR = 1.17, 95% CI: 1.03–1.32, *p* = 0.016), suggesting that BMI may be a non‐negligible influencing factor when other factors are comprehensively considered.

PSA screening can significantly reduce the metastasis and mortality of PCa [25]. However, since prostatic inflammation and BPH can also elevate serum PSA levels, leading to a lack of specificity, there is a risk of overdiagnosis [26]. We stratified PSA into three groups to improve its specificity for csPCa. The results showed that PSA ≥ 10 was an independent predictor of csPCa. When PSA was in the 4–10 ng/mL range, neither univariate nor multivariate analysis showed a significant association with csPCa, possibly because PSA in this range lacks sufficient tumor specificity and is easily confounded by other factors [27]. Similarly, univariate analysis showed that an elevated fPSA was associated with an increased risk of csPCa (OR = 1.10, 95% CI: 1.06–1.15, *p* < 0.001), but it could not independently predict csPCa. Currently, the value of PSA and its derivatives in the diagnosis of csPCa remains controversial [28, 29]. However, most studies suggest that PSA‐related indicators combined with imaging and other clinical parameters can actively monitor the risk of csPCa and prognosis [30, 31].

Our results showed that the detection rates of csPCa in S‐PI‐RADS category 4 and 5 lesions were both high, at 31.8% and 68.8%, respectively. By contrast, the csPCa detection rate in S‐PI‐RADS category 3 lesions (13.8%) was significantly lower than that in categories 4 and 5. This finding is consistent with other studies confirming the generally low incidence of csPCa in PI‐RADS category 3 lesions [32, 33]. A recent study has demonstrated that PI‐RADS scores have high application value in diagnosing csPCa [34]. Scores of 4–5 indicate a high probability of clinically significant cancer, and biopsy should be considered to confirm the diagnosis [35]. Our results also showed that S‐PI‐RADS scores of 4 and 5 were independent predictors of csPCa, with higher S‐PI‐RADS scores demonstrating significant advantages in csPCa diagnosis. This study used an AI‐assisted simplified S‐PI‐RADS scoring system based on DWI and T2WI. Correlation analysis between S‐PI‐RADS grading and ISUP in the PCa group showed a significant moderate correlation between S‐PI‐RADS and ISUP (*r* = 0.44, *p* < 0.001), verifying the noninvasive predictive ability of the S‐PI‐RADS system for the pathological characteristics of prostate cancer. Some studies have also confirmed that the S‐PI‐RADS score exhibits performance comparable to or superior to that of standard mp‐MRI in PCa diagnosis, while offering advantages such as no need for contrast agents, shortened scanning time, and reduced examination costs [11, 36]. By simplifying the S‐PI‐RADS scoring process with AI, this study not only validated the clinical value of S‐PI‐RADS in prostate cancer diagnosis but also provided a new paradigm for constructing an “efficient, economical, and precise” imaging evaluation system for prostate cancer. Meanwhile, prostate volume was obtained using AI software. Consistent with previous findings [37], we found that PV was an independent risk factor for csPCa (OR = 0.97, 95% CI: 0.96–0.99, *p* < 0.001). Larger prostate volume was associated with a lower risk of csPCa, which may be attributed to prostate volume enlargement being more commonly caused by factors such as benign prostatic hyperplasia.

The European Association of Urology's guidelines for PCa diagnosis and treatment encourage more research to incorporate MRI quantitative indicators into the scoring system. In our study, we introduced a noninvasive quantitative measurement of intraprostatic iron content based on magnetic resonance R2* imaging. The results of this study showed that the csPCa group exhibited higher R2* value than the ncsPCa group. Early magnetic resonance studies on iron deposition in tissues primarily focused on using spin echo (SE) T2* imaging techniques. T2* values can effectively reflect the influence of tissue magnetic susceptibility on image signals, and their applications in the prostate have become increasingly widespread [38, 39]. The results of D W et al.'s study showed that T2* values based on T2* mapping exhibited good value in differentiating prostate cancer from BPH (AUC = 0.865, *p* < 0.001) and distinguishing PCa patients with ISUP ≤ 2 from those with ISUP > 2 (AUC = 0.867, *p* < 0.001), which can reflect tumor iron metabolism [16]. To visually quantify iron content, the R2* value was introduced. Notably, R2* is the reciprocal of T2 (R2* = 1/T2*), and both metrics can reflect changes in tissue magnetic susceptibility induced by iron deposition. However, R2* shows a positive linear response to changes in iron concentration, making it more intuitive to interpret and more convenient for statistical analysis in quantitative research compared to T2*. When tissue iron content increases, paramagnetic iron compounds disrupt the uniformity of the local magnetic field, leading to an increase in tissue R2*. The R2* mapping used in this study was based on a T2*‐corrected three‐dimensional multi‐echo Dixon sequence, which features fast imaging speed, high signal‐to‐noise ratio, and extremely high sensitivity in detecting changes in the local magnetic environment. The results of this study showed that multiple logistic regression analysis identified increased R2* values as a statistically significant independent predictor of high‐risk csPCa (OR = 1.17, 95% CI: 1.10–1.25; *p* < 0.001). In PCa patients, the accelerated proliferation of malignant tumor cells relies on high metabolism and high nutritional requirements to promote growth and metastasis. As a key nutrient, iron plays a critical role in angiogenesis and tumor metastasis, significantly facilitating cancer cell growth and invasive ability [15, 16]. Additionally, the rapid proliferation of tumor cells leads to insufficient local tissue oxygen supply in the tumor, creating a hypoxic microenvironment, which may increase the content of deoxyhemoglobin. At the same time, the proliferation of malignant epithelial cells causes disordered and disrupted glandular structures, reducing glandular lumens and relative water content, leading to microcirculatory disorders, which further increase deoxyhemoglobin [40, 41]. Deoxyhemoglobin is paramagnetic, causing an increase in R2* values. From a biological standpoint, the malignant proliferation of prostate cancer drives two key pathological changes: first, the high metabolic demand of tumor cells induces iron overload, where paramagnetic iron ions directly disrupt the homogeneity of the local magnetic field; second, the hypoxic microenvironment resulting from rapid tumor growth promotes deoxyhemoglobin accumulation, which further amplifies magnetic field inhomogeneities. These combined factors lead to increased R2* values in the csPCa group. Therefore, R2* values can provide additional information from the perspectives of tumor metabolism and microenvironment and serve as potential predictive indicators for prostate cancer‐related events. In this study, we constructed a predictive model for csPCa integrating AI‐assisted S‐PI‐RADS, R2* mapping, and clinical indicators. With an AUC of 0.915, this model significantly outperforms traditional models relying solely on imaging features or clinical parameters, providing a novel non‐invasive diagnostic approach for csPCa.

A nomogram is a graphical tool converting multiple predictive indicators into intuitive scale lines via mathematical models to provide individualized quantitative risk predictions. It is simple and easy to understand, making it suitable for clinical personalized assessment. In this study, a novel nomogram based on BMI, PSA, PV, S‐PI‐RADS, and R2* was developed and validated, which effectively distinguished patients with csPCa from those with ncsPCa (AUC = 0.915, sensitivity = 85.2%, specificity = 80.9%). Decision curve results showed that the combined model had the optimal clinical net benefit. In recent years, numerous studies have reported that nomogram models combining clinical and imaging indicators can be used to predict csPCa. Hiremath A et al. developed a nomogram integrating deep learning‐derived imaging features (including ADC maps), PI‐RADS scores, and clinical variables to distinguish csPCa from ncsPCa, with an AUC of 0.81 in the independent validation cohort [42]. Cheng et al. constructed a nomogram model incorporating age, PSAD, and PI‐RADS v2.1 scores, and decision curve analysis showed that the net benefit of this model was significantly higher than using PI‐RADS v2.1 scores or PSAD alone, indicating good clinical efficacy [43]. These findings are consistent with those of the present study, both confirming that combined predictive models can significantly enhance the predictive efficacy for csPCa. In our study, adding the R2* value to the baseline model further enhanced the predictive efficacy for csPCa (AUC = 0.915 vs. 0.891, *p* < 0.05), further demonstrating that integrating imaging and clinical indicators can effectively discriminate between csPCa and ncsPCa. As a non‐invasive indicator quantifying tumor iron metabolism, R2* mapping supplements the limitations of traditional morphological assessment from the perspective of the tumor microenvironment, emerging as a key factor contributing to the improved performance of the present model. Notably, differences in technical pathways and core objectives among various studies provide diverse perspectives for csPCa diagnosis. Vescovo et al. [44] emphasized that sample quality and standardized procedures are the core of reliable research. Their multicenter study on BRCA1/2 gene sequencing in prostate cancer showed that sample storage time, type, and DNA quality significantly affect the detection success rate. We performed imaging analysis through an automated AI workflow to reduce human variability, which is highly consistent with the core idea of this study. While our study lacked multicenter external validation, the standardized AI‐driven analytical pipeline ensures result reproducibility, laying a foundation for future multicenter verification to enhance generalizability. Santucci et al. [45] focused on predicting prostate cancer lymph node involvement (LNI), utilizing radiomic features from mp‐MRI (T2WI/DWI/ADC) combined with clinical nomograms. Their optimal random forest model achieved an AUC of 0.89, but relied on manual lesion segmentation and lacked quantitative indicators for the tumor microenvironment, differing from the core objective of non‐invasive csPCa diagnosis in our study. Prata et al. [46] also targeted csPCa prediction, but their model had an AUC of only 0.804, relied on manual PI‐RADS scoring, and lacked metabolism‐related quantitative parameters. In contrast, our model avoids subjective bias through AI‐driven S‐PI‐RADS, further enhances discriminative efficacy with the integration of R2* mapping, and reduces examination costs and patient burden via a simplified bp‐MRI protocol that eliminates the need for DCE sequences. To facilitate clinical practice, we have developed a web‐based dynamic online nomogram that allows doctors to interactively operate on the webpage. By adjusting the values of the predictors and clicking the “Predict” button on the webpage, the changes in the predicted results can be quickly calculated. This approach helps to personalize the prediction of csPCa occurrence probability in combination with the actual clinical patients, assisting clinicians in formulating individualized treatment plans. In clinical practice, our model can serve as a refined supplementary tool for current diagnostic workflows (e.g., those recommended by the European Association of Urology [EAU]/National Comprehensive Cancer Network [NCCN]). For S‐PI‐RADS 3 “gray zone” cases, our dynamic online nomogram can rapidly integrate the patient's BMI, PSA value, prostate volume, AI‐S‐PI‐RADS score, and R2* value to calculate an individualized predicted probability of csPCa. By setting a reasonable risk threshold (such as the threshold corresponding to the Youden index in this study, or adjusted based on clinical preferences), the model helps further stratify csPCa risk and reduce unnecessary biopsies. Future studies need to validate its effectiveness in reducing biopsy rates and cost‐effectiveness in real‐world clinical settings.

Our study has several limitations. First, as a single‐center study, the constructed nomogram model has only undergone internal validation (Bootstrap method). Although internal validation demonstrated good calibration and discrimination (C‐index = 0.887), relying solely on internal validation is insufficient to fully verify the model's generalization ability, and external multicenter validation remains an essential step. Differences exist in MRI equipment parameters, examination procedures, post‐processing pipelines, and baseline characteristics of patient populations across centers that may affect the stability of the model's performance, particularly for quantitative metrics like R2* mapping. In the future, it is necessary to collaborate with multiple medical institutions to conduct large‐sample external validation, further confirming the model's applicability in different clinical scenarios. Second, there is a certain degree of class imbalance between csPCa (115 cases) and ncsPCa (230 cases) in the sample, which may lead to prediction bias of the model toward csPCa. Although this issue has been alleviated to some extent through Bootstrap validation, it may still affect the model's stability in real clinical settings. Meanwhile, the overall sample size is relatively limited, and the data scale of some subgroups is small, which may restrict the model's predictive efficacy for special populations (such as elderly patients or those with comorbidities), thereby influencing the robustness of the results. Finally, some pathological results are based on needle biopsy rather than radical prostatectomy specimens, which may lead to lesion sampling bias.

## 5 Conclusion

This study introduces a non‐invasive quantitative method for measuring iron content, confirms the clinical utility of R2* in predicting csPCa, and constructs a csPCa prediction model by integrating clinical and imaging parameters, providing a novel, more efficient, and simplified risk assessment tool.

## Author Contributions

**Xin Li:** conceptualization (equal), data curation (equal), formal analysis (equal), methodology (equal), writing – original draft (lead). **Yonggui Shi:** conceptualization (equal), formal analysis (equal), investigation (equal), methodology (equal), writing – original draft (equal). **Jing Fang:** data curation (equal), formal analysis (equal), methodology (equal), software (equal), visualization (equal).**Rong Zhang:** conceptualization (equal), formal analysis (equal), visualization (equal). **Xiaojing He:** funding acquisition (equal), resources (equal), supervision (equal), writing – review and editing (supporting). **Guangyong Ai:** conceptualization (equal), formal analysis (equal), methodology (equal), resources (equal), supervision (equal), visualization (equal), writing – original draft (equal), writing – review and editing (lead).

## Funding

This work was supported by Chongqing Natural Science Foundation (CSTB2024NSCQMSX0616), Science and Health Joint Medical Research Project of Chongqing (2024ZDXM004), Senior Medical Talents program of Chongqing for Young and Middle aged, Kuanren Talents Program of the second affiliated hospital of Chongqing Medical University, Program for Youth Innovation in Future Medicine, Chongqing Medical University, Engineering Research Center for Fundamental and Translational Intelligent Molecular Imaging, Chongqing Municipal Education Commission and Key Laboratory of Intelligent Processing and Applications of Medical Imaging Big Data, Chongqing Municipal Health Commission.

## Conflicts of Interest

The authors declare no conflicts of interest.

## Supporting information


**Table S1:** MR imaging parameters.
**Table S2:** S‐PI‐RADS based on bpMRI.

## Data Availability

Data available on request from the authors.
