# Supplementary material for: Development and Validation of an AI‐Assisted Predictive Model Integrating R2* Mapping and Clinical Indicators for Clinically Significant Prostate Cancer
Source: Cancer Med. 2026 Feb 18;15(2):e71656. doi: 10.1002/cam4.71656 (PMC12916443; doi:10.1002/cam4.71656)
Supplement: Supplementary file 1 — Table S1: MR imaging parameters. Table S2: S‐PI‐RADS based on bpMRI. [file CAM4-15-e71656-s001.docx]

**Supplementary Table S1. MR imaging parameters.**

| **Sequence** | **Repetition time**  **(ms)** | **Echo time**  **(ms)** | **Slice thickness**  **(mm)** | **Slice gap**  **(mm)** | **Field of View**  **(cm^2^)** | **Scan matrix** | **Averages** | **Additional Features** |
| --- | --- | --- | --- | --- | --- | --- | --- | --- |
| T2WI_qtse_tra | 3290 | 77 | 3.0 | 0 | 20 x 20 | 320 x 240 | 2 | TF = 17 |
| T2WI_qtse_fs_tra | 3290 | 77 | 3.0 | 0 | 20 x 20 | 320 x 240 | 2 | TF = 17 |
| T2WI_qtse_cor | 3600 | 88 | 3.0 | 0 | 20 x 20 | 384 x 288 | 1 | TF = 20 |
| T2WI_qtse_sag | 3800 | 110 | 3.0 | 0 | 24 x 24 | 320 x 224 | 2 | TF = 20 |
| Resolve_diff_b0_1400_tra | 3800 | 84 | 3.0 | 0 | 20 x 20 | 118 x 118 | 2 | b = 0/1400 |
| q-Dixon | 9.14 | 1.28 , 2.53, 3.78, 5.03, 6.28, and 7.53 | 3.0 | 0 | 25 x 20 | 160 x 128 | 1 | FA = 4° |

Abbreviations: Tra, transverse; Cor, coronal; Sag, sagittal; Diff, diffusion; qtse, quiet turbo-spin-echo; fs, fat suppressed; q-Dixon, T2*-corrected 3D multi-echo Dixon; TF, Turbo factor; FA, flip angle; T2WI, T2-weighted imaging.

**Supplementary Table S2. S-PI-RADS based on bpMRI.**

| **PZ** | | |  | **TZ** | | |
| --- | --- | --- | --- | --- | --- | --- |
| **DWI** | **T2WI** | **Total score** |  | **T2WI** | **DWI** | **Total score** |
| 1 | Any | 1 |  | 1 | Any | 1 |
| 2 | Any | 2 |  | 2 | ≤3 | 2 |
| 3 | Any | 3 |  | 2 | ≥4 | 3 |
| 4 | Any | 4 |  | 3 | ≤4 | 3 |
| 5 | Any | 5 |  | 3 | 5 | 4 |
|  |  |  |  | 4 | Any | 4 |
|  |  |  |  | 5 | Any | 5 |

Abbreviations: S-PI-RADS, Simplified Prostate Imaging Reporting and Data System; bpMRI, biparametric magnetic resonance imaging; PZ, Peripheral Zone ; TZ, Transition Zone; DWI, diffusion-weighted imaging; T2WI, T2-weighted imaging.
